# Interactive and Independent Associations between the Socioeconomic and Objective Built Environment on the Neighbourhood Level and Individual Health: A Systematic Review of Multilevel Studies

**DOI:** 10.1371/journal.pone.0123456

**Published:** 2015-04-07

**Authors:** Steffen Andreas Schüle, Gabriele Bolte

**Affiliations:** Department of Social Epidemiology, Institute of Public Health and Nursing Research, University of Bremen, Bremen, Germany; Hamamatsu University School of Medicine, JAPAN

## Abstract

**Background:**

The research question how contextual factors of neighbourhood environments influence individual health has gained increasing attention in public health research. Both socioeconomic neighbourhood characteristics and factors of the built environment play an important role for health and health-related behaviours. However, their reciprocal relationships have not been systematically reviewed so far. This systematic review aims to identify studies applying a multilevel modelling approach which consider both neighbourhood socioeconomic position (SEP) and factors of the objective built environment simultaneously in order to disentangle their independent and interactive effects on individual health.

**Methods:**

The three databases PubMed, PsycINFO, and Web of Science were systematically searched with terms for title and abstract screening. Grey literature was not included. Observational studies from USA, Canada, Australia, New Zealand, and Western European countries were considered which analysed simultaneously factors of neighbourhood SEP and the objective built environment with a multilevel modelling approach. Adjustment for individual SEP was a further inclusion criterion.

**Results:**

Thirty-three studies were included in qualitative synthesis. Twenty-two studies showed an independent association between characteristics of neighbourhood SEP or the built environment and individual health outcomes or health-related behaviours. Twenty-one studies found cross-level or within-level interactions either between neighbourhood SEP and the built environment, or between neighbourhood SEP or the built environment and individual characteristics, such as sex, individual SEP or ethnicity. Due to the large variation of study design and heterogeneous reporting of results the identification of consistent findings was problematic and made quantitative analysis not possible.

**Conclusions:**

There is a need for studies considering multiple neighbourhood dimensions and applying multilevel modelling in order to clarify their causal relationship towards individual health. Especially, more studies using comparable characteristics of neighbourhood SEP and the objective built environment and analysing interactive effects are necessary to disentangle health impacts and identify vulnerable neighbourhoods and population groups.

## Background

Since the late 1990s an increasing number of epidemiological studies have analysed whether the socioeconomic, built, social or ethnic neighbourhood environment have an independent effect on individual health outcomes or health-related behaviours [1, 2]. There is an overall conclusion that underlying mechanisms of the association between neighbourhood environments and health are quite complex and both mediating and interacting mechanisms should be considered. Therefore, various conceptual models were developed describing pathways explaining associations between neighbourhood context and individual health [2–9].

For a better systematization of possible connections between neighbourhood characteristics and individual health a distinction between compositional and contextual effects is widely established in the literature. A compositional effect is present if health differences between neighbourhoods are attributed to individual characteristics, the so-called composition of neighbourhood residents, such as individual health behaviours, health status or individual socioeconomic position (SEP). The term contextual effect is used if variables at the neighbourhood level, such as features of the built or social environment, have an effect on individual health outcomes while adjusting for possible confounders at the individual level to avoid an ecological fallacy [10–12]. This more abstract distinction has also been discussed critically in the literature [13]. However, it provides a good basis for suggesting conceptual pathways in which ways neighbourhood context can affect individual health.

To separate out potential contextual neighbourhood effects from individual effects a multilevel modelling approach is an appropriate analytic strategy addressing such issues. Multilevel modelling offers the possibility to sort out how much variance of health outcomes between neighbourhoods is related to individual factors and how much is explained by contextual factors on the neighbourhood level. A multilevel model combines data on at least two hierarchical levels: aggregated variables on the neighbourhood level (2nd level) and variables on the individual level from residents within the neighbourhood (1st level). Thus, simultaneous examinations of independent effects of each level and interactions within and across levels on individual health outcomes are possible while accounting for the potential dependency of individual observations sharing the same characteristics of higher level variables [11, 14].

Systematic reviews showed that most neighbourhood studies focused on factors of neighbourhood SEP from aggregated census data. They analysed whether neighbourhood SEP has a contextual effect on individual health while simultaneously adjusting for individual socio-demographic characteristics. Many of these studies found out that a low neighbourhood SEP was independently associated with poor health, such as increased mortality risk, poor self-rated health, depressive symptoms, low birth weight or cardiovascular risk factors [15–22]. Evidence from these studies raised the question which underlying factors explain independent associations between neighbourhood SEP and individual health. Many studies hypothesized that poor neighbourhoods are exposed to a poor built environment, such as air pollution, lack of green space or an unhealthy food environment. Thus, an integrated consideration of neighbourhood SEP, built environmental factors and socioeconomic factors on the individual level is needed to explore underlying mechanisms how neighbourhood SEP and the built environment are connected and associated with health.

The term ‘built environment’ can be systematically differentiated from the term ‘natural environment’. Both terms belong to the physical environment. Schulz and Northridge define the built environment as that part of the physical environment which *“encompass all of the buildings*, *spaces*, *and products that are created or significantly modified by people (…)”* (Page 456) [23]. In urban environments none of the environment is natural because even parks including natural components, such as green space or water, are to some extent created or modified by people, and can be assigned to the built environment, too. Thus, the built environment covers many dimensions in an urban context, such as land use, transportation systems, services, public resources, zoning regulations or building characteristics [24]. The built environment can be measured subjectively or objectively. Subjective measures are mostly self-reported perceptions conducted in survey questionnaires. Objective measures can be either collected in the field or obtained from existing land use data in Geographic Information Systems (GIS). Systematic reviews focusing on the evidence how factors of the built environment influence health indicated the increasing importance of this neighbourhood dimension [25–29]. These reviews considered primarily cardiovascular risk factors, such as overweight or low physical activity. Though studies of the built environment gave partly inconsistent results, all reviews concluded that the built environment can significantly impact individual health.

The links between neighbourhood SEP and exposures from the built environment and health are captured by the environmental justice framework. A conceptual model derived from this framework contains two main hypothetical pathways how socioeconomic position, environmental exposures and health are connected: The first hypothesis states that environmental exposures are social unequally distributed (exposure variation by SEP), the second hypothesis states that neighbourhoods or individuals with a low SEP are more vulnerable to environmental exposures [30].

Both neighbourhood SEP and built environmental factors play a significant role for explaining health inequalities between neighbourhoods. However, to the best of our knowledge a systematic review focusing on to what extent both characteristics of neighbourhood SEP and the built environment are simultaneously considered in epidemiological neighbourhood studies, and how they interact with each other or with individual characteristics has not been carried out so far.

The overall goal of this systematic review is to identify epidemiological studies with a multilevel modelling approach considering characteristics of neighbourhood SEP and the objective built environment simultaneously in order to disentangle their independent or interactive effects on individual health outcomes.

The primary research questions is, how characteristics of neighbourhood SEP and the objective built environment are associated with individual health outcomes or health-related behaviours if both dimensions are considered simultaneously in multilevel modelling. Secondary, the review summarizes knowledge on interactions between neighbourhood SEP, the built environment and individual SEP.

## Methods

The three databases PubMed, PsycINFO, and Web of Science were searched on the 5^th^ of November 2013. The research question, search strategy and inclusion criteria were developed before the review process. There is no registered protocol reference number, however. Search terms were generated for title and abstract screening in order to identify neighbourhood studies with a multilevel modelling approach considering both socioeconomic and built environmental factors. In order to identify synonyms for the terms ‘neighbourhood’, ‘built environment’, ‘socioeconomic environment’ and ‘multilevel modelling’, the terminology in already existing reviews and their cited studies dealing with these topics were additionally considered. In PubMed terms of Medical Subject Headings were taken into account (Table 1). Title and abstracts were screened by two reviewers independently with predefined inclusion criteria. A third reviewer was consulted if there was disagreement. If one of the inclusion criteria could not be clearly identified in the abstract, the full text of the record was analysed for eligibility by one reviewer. An explicit search on grey literature was not performed because the review focused on observational epidemiological studies applying advanced statistical modelling which are most likely to be found in scientific journals. However, to take into account potential publication bias, we did not limit our analysis on papers published in peer-reviewed journals. References of finally included records were additionally checked. Neighbourhood studies applying a multilevel modelling approach are a relatively recent study type. Therefore, we did not restrict our search to a specific time period.

**Table 1:** Search terms and Medical Subject Headings in PubMed.

Search	Query
#1	neighborhood [Title/Abstract] OR neighbourhood [Title/Abstract] OR area [Title/Abstract] OR place [Title/Abstract] OR residence [Title/Abstract] OR community [Title/Abstract] OR region [Title/Abstract]
#2	multilevel [Title/Abstract] OR multi-level [Title/Abstract] OR hierarch* [Title/Abstract] OR "multilevel analysis" [MeSH Terms] OR "Small-Area Analysis" [MeSH Terms] OR "mixed effect*" [Title/Abstract] OR "random effect*" [Title/Abstract]
#3	"social environment*" [Title/Abstract] OR socioeconomic [Title/Abstract] OR socio-economic [Title/Abstract] OR sociodemographic [Title/Abstract] or socio-demographic [Title/Abstract] OR "social environment" [MeSH Terms] or "socioeconomic factors" [MeSH Terms]
#4	"physical environment*" [Title/Abstract] OR built [Title/Abstract] OR build* [Title/Abstract] OR "living environment*" [Title/Abstract] OR housing [Title/Abstract] OR pollution [Title/Abstract] OR burden* [Title/Abstract]
Final search	#1 AND #2 AND #3 AND #4

As suggested by Krieger et al. the term ‘socioeconomic position’ (SEP) is used. The term ‘SEP’ combines actual economic and social resources with prestige-based characteristics which relatively position individuals, households and neighbourhoods in society [31, 32].

The following inclusion criteria were applied:

1. Observational studies applying multilevel modelling and considering factors of the neighbourhood environment as higher level variables. Studies focusing exclusively on other environments were excluded, such as the school or work environment. Moreover, studies taking into account subjects from clinical settings or focusing on study populations with health problems were also excluded. Clinical trials and intervention studies were excluded, too.
2. Studies from USA, Canada, Australia, New Zealand and Western European Countries
3. Physical or mental health outcomes, or health-related behaviours measured at the individual level.
4. Simultaneous consideration of at least one characteristic of neighbourhood SEP from the whole neighbourhood population and at least one objective measure of the built environment in one multilevel model. Studies were excluded if neighbourhood SEP was only considered as an adjustment variable.
5. Measures of the objective built environment. Papers were excluded which considered only measures of the perceived built environment. Studies showed that there is low to moderate agreement between objective and perceived measures of the built environment [33, 34]. Moreover, studies which assessed the neighbourhood built environment via observational methods with trained staff were also excluded due to limitations in validity [35].
6. Adjustment for at least one individual socioeconomic factor. Ethnicity alone was not considered as a sufficient indicator for socioeconomic position [36]. Therefore, studies considering only specific ethnic population samples were also excluded.

Each included study was described in a summary table and coded related to: outcome, population sample, country, considered factors of neighbourhood SEP and the objective built environment, and individual and further contextual factors. Because the review focused on neighbourhood SEP and the objective built environment, other considered neighbourhood characteristics in the study, such as measures of crime, social capital, residential stability, perceived built environment or segregation were indicated also in the last column (Table 2).

**Table 2:** Description of studies.

Reference	Health outcomes	Sample	Country	Design	Neighbourhood SEP	Objective built environment	Individual and further contextual factors
	**Physical activity, overweight, quality of life, and depressive symptoms**						
De Meester, 2012 [46]	**1.** Average activity level in counts per minute and moderate-to-vigorous physical activity in mean minutes per day assessed with accelerometer (continuous); **2.** Reported walking, cycling, and sport during leisure time, and active transport to and from school in minutes per day (continuous)	Adole-scents (13–15 years); N = 637	Belgium	Cross-sectional	Median annual household income	Walkability index (residential density, intersection density, land use mix)	**Individual:** educational attainment and employment status of parents.
Owen, 2007 [48]	**1.** Reported walking for transport in minutes per week and number of days past week (continuous); **2.** Reported walking for recreation in minutes per week and number of days past week (continuous)	Adults (20–65 years); N = 2,650	Australia	Cross-sectional	Median annual household income	Walkability index (dwelling density, street connectivity, land use mix, net retail area)	**Individual:** age, sex, education, annual household income, children in household, reported reasons for neighbourhood self-selection.
Prince, 2012 [55]	**1.** Reported physical activity (dichotomous): inactive and moderately physical activity vs. high physical activity; **2.** Under-/normal weight vs. overweight/obesity (dichotomous)	Adults (≥18 years); N = 4,727	Canada	Cross-sectional	Index (households below the low-income cut-off, average household income, unemployed residents, residents with less than a high school education, single-parent families)	Number of winter indoor/outdoor facilities and summer outdoor facilities per 1,000 residents; green space and park area (km²); bike/walking path length (km); number of grocery stores, fast food outlets, convenience stores, restaurants, and speciality food stores per 1,000 residents	**Individual:** age, sex, education, household income, smoking status, season of data collection, community belonging. **Contextual:** councillor voting rates, crime, season.
Prince, 2011 [56]	**1.** Reported physical activity (dichotomous): inactive and moderately physical activity vs. high physical activity; **2.** Under-/normal weight vs. overweight/obesity (dichotomous)	Adults (≥18 years); N = 3,514	Canada	Cross-sectional	Index (households below the low-income cut-off, average household income, unemployed residents, residents with less than a high school education, single-parent families)	Number of winter indoor/outdoor facilities and summer outdoor facilities per 1,000 residents; green space and park area (km²); bike/walking path length (km); number of grocery stores, fast food outlets, convenience stores, restaurants, and speciality food stores per 1,000 residents	**Individual:** age, sex, education, household income, smoking status, community belonging. **Contextual:** councillor voting rates, crime, season.
Riva, 2009 [44]	Reported number of 10-minute episodes walking in the last seven days (continuous): walking for any motive, utilitarian walking, recreational walking	Adults (≥45 years); N = 2,923	Canada	Cross-sectional	Percentage of residents with a university education	Urbanity index (population density, land use mix, and accessibility to services)	**Individual:** age, sex, education.
Sallis, 2009 [59]	**1.** Moderate-to-vigorous physical activity in minutes per day assessed with accelerometer (continuous); **2.** Reported walking for leisure and transportation (continuous): minutes per week; **3.** BMI (continuous); **4.** Overweight or obesity (dichotomous); **5.** Obesity (dichotomous); **6.** Reported physical quality of life (continuous); **7.** Reported mental quality of life (continuous); **8.** Reported depressive symptoms (continuous)	Adults (20–65 years); N = 2,199	USA	Cross-sectional	Median annual household income	Walkability index (residential density, retail floor area ratio, mixed land use, intersection density)	**Individual:** age, sex, education, ethnicity, number of adults and motor vehicles in household, marital status, number of people in household, number of years living at current address, reported reasons for moving to neighbourhood.
Scott, 2009 [57]	**1.** Reported number of days per week walking to school, to work, to a store, to the bus, to do an errand, or to a neighbour’s house (continuous); **2.** Reported number of days per week walking outdoors just for exercise or pleasure (continuous); **3.** BMI (continuous)	Adults (≥18 years); N = 1,815	USA	Cross-sectional	Index (adults older than 25 with less than a high school education, male unemployment, households with income below the poverty line, households receiving public assistance, households with children headed only by a female, median household income)	Number of parks and markets in a one mile radius around home address, street connectivity, median block length, street density	**Individual:** age, sex, ethnicity, income, access to car in the household, perceived neighbourhood safety, utilitarian and recreational walking considered as an adjustment variables in the final BMI model.
Slater, 2010 [58]	**1.** Reported vigorous exercise (dichotomous); **2.** Reported sports participation (dichotomous); **3.** Reported physical activity participation (dichotomous); **4.** BMI (continuous); **5.** Obesity (dichotomous)	Students (13–16 years); N = 10,620–36,929	USA	Cross-sectional	Median annual household income	Number of physical activity outlets per 10,000 residents, ratio of higher road classes to all other roads, compactness index (residential density and street connectivity)	**Individual:** sex, grade, ethnicity, parental education, student income, students work, mother´s work status, private or public school, region, year of data collection, perceived environment (physical disorder scale, physical activity setting scale, perceived neighbourhood safety. **Contextual:** community observations by trained field teams (advertising, recreational space, social interactions, events, safety, general upkeep of the area).
Sundquist, 2011 [45]	**1.** Moderate-to-vigorous physical activity in minutes per day assessed with accelerometer (continuous); **2.** Reported walking for active transportation and leisure (continuous and dichotomous)	Adults (20–66 years); N = 2,269	Sweden	Cross-sectional	Median family income	Walkability index (residential density, street connectivity, land use mix)	**Individual:** age, sex, family income, marital status.
Van Dyck, 2010 [42]	**1.** Sedentary time assessed with accelerometer: percentage of wearing time below 100 counts per minute (continuous); **2.** Reported sitting time in the past 7 days in minutes per day (continuous)	Adults (20–65 years); N = 1,200	Belgium	Cross-sectional	Median annual household income	Walkability index (residential density, street connectivity, land use mix)	**Individual:** sex, age, education, employment status, occupation, living with children.
Van Dyck, 2010 [43]	**1.** Moderate-to-vigorous physical activity in minutes per day assessed with accelerometer (continuous); **2.** Reported walking, recreation, and cycling for transport, and motorized transport in minutes per week (continuous)	Adults (20–65 years); N = 1,166	Belgium	Cross-sectional	Median annual household income	Walkability index (residential density, street connectivity, land use mix)	**Individual:** age, sex, education, working status, BMI.
Wen, 2009 [47]	**1.** Reported number of weekly workout/exercise (dichotomous): one to three times vs. four times or more; **2.** Reported regular exercise past year (dichotomous)	Adults (≥18 years); N = 3,530	USA	Cross-sectional	Index (households with an annual income >$50,000, families below the poverty line, residents ≥25 years with college education, female-headed households, households on public assistance, neighbourly trust, norms of reciprocity, violence)	Distance to subway and parks from the tract centroid; land use mix; number of art centres, cultural institutions, leisure venues, and entertainment facilities in a three-mile buffer from the tract centroid; number of restaurants and bars in a one mile-buffer; number of libraries, churches, and educational institutions in a two-mile buffer; number of health and human services in a three-mile buffer	**Individual:** age, sex, ethnicity, marital status, education, household income. **Contextual:** pedestrian injuries per 100,000 persons, residential density.
Inagami, 2009 [54]	BMI (continuous)	Adults (≥18 years); N = 2,156	USA	Cross-sectional	Index (residents below the poverty line, households headed by women, unemployed male residents, families on public assistance)	Number of fast food outlets and number of total food outlets divided by census tract roadway miles	**Individual:** age, sex, education, ethnicity, employment, marital status, annual household income, immigrant status, car ownership.
Moore, 2013 [49]	BMI (continuous)	Adults (45–84 years); N = 1,503	USA	Cross-sectional	Index (Sixteen variables of education, occupation, income, and housing value)	Density of recreational facilities and healthy food environments in a one mile buffer around home address	**Individual:** age, sex, ethnicity, education, household income, perceived neighbourhood environment (aesthetic quality, walking environment, healthy food availability), perceived neighbourhood safety and social cohesion.
Ross, 2007 [53]	BMI (continuous)	Adults (20–64 years); N = 32,964	Canada	Cross-sectional	Percentage of residents with low education, median household income	Dwelling density (dwellings per square kilometre)	**Individual:** age, sex, income, education, marital status, smoking status, work-related physical activity, fruit/vegetable consumption, daily stress, immigrant status. **Contextual:** percentage of recent immigrants.
Wang, 2007 [51]	BMI (continuous)	Adults (25–74 years); N = 7,595	USA	Cross-sectional	Index (median family income, median housing value, blue collar workers, unemployed residents, residents having less than high school education)	Total number of stores and fast food restaurants divided by neighbourhood size including a half mile buffer zone around the neighbourhood; proximity to food store or fast food restaurant from home address	**Individual:** age, sex, ethnicity, individual SEP index (household income and educational attainment), smoking status, physical activity, nutrition knowledge.
Matthews, 2010 [66]	Composite health score (continuous): presence of any of six physical health problems and self-rated health (higher values indicate better health).	Adults (≥18 years); N = 4,093	USA	Cross-sectional	Index I (resident/room ratio, female-headed households, unemployment rate, poverty, people receiving public assistance); index II (residents with at least a bachelor´s degree, managerial or professional occupations)	Daily vehicle miles travelled, toxic release inventory sites, residual waste operations facilities, medical resources index (licensed and staffed beds, licensed medical doctors, hospitals, patients ≥65 years receiving flu vaccine)	**Individual:** age, sex, ethnicity, stress level, marital status, employment status, retired, incapable of working, education, poverty status, religious service attendance, insurance and dental insurance, regular source of care, transportation difficulty in seeing a doctor, neighbourhood participation and trust. **Contextual:** residential stability, safety index (violent crimes, property crimes, missing persons).
Yang, 2010 [70]	Self-rated day-to-day stress on a scale from 1 to 10 (continuous): higher value indicate more stress	Adults (≥18 years); N = 4,095	USA	Cross-sectional	Index (female headed households, unemployment rate, poverty, residents receiving public assistance, median household income, residents with at least a bachelor´s degree)	Daily vehicle miles travelled based on length of road and average daily traffic estimate; toxic release inventory sites and residual waste operation sites	**Individual:** age, sex, ethnicity, marital status, employment status, education, poverty, food insecurity, health score (calculated from reported physical health problems and self-rated health), religiosity, trust in neighbourhood people. **Contextual:** crime, residential stability.
Dragano, 2009 [73]	Objective coronary artery calcification (dichotomous)	Adults (45–75 years); N = 4,301	Germany	Cross-sectional	Unemployment rate	Individual distance to major road from home address (>100 m and ≤100 m)	**Individual:** age, education.
Dragano, 2009 [74]	Objective coronary artery calcification (dichotomous)	Adults (45–75 years); N = 4,301	Germany	Cross-sectional	Unemployment rate	Individual distance to major road from home address (0–50 m, 51–100 m, 101–200 m, ≥200 m)	**Individual:** age, education, economic activity, smoking, physical inactivity, overweight, hypertension, total cholesterol.
Chuang, 2005 [71]	Reported number of smoked cigarettes on average per day (continuous)	Adults (25–74 years); N = 8,121	USA	Cross-sectional	Index (residents with less than high school education, blue collar workers, unemployed residents, median annual family income, median housing value)	Number of convenience stores per square mile, individual distance to nearest convenience store from home address, number of convenience stores in a one mile radius around home address	**Individual:** age, sex, ethnicity, individual SEP index (education, poverty status based on federal poverty threshold).
Pollack, 2005 [72]	Reported alcohol consumption (dichotomous): heavy alcohol consumption (>7 drinks per week for females; >14 drinks per week for males)	Adults (25–74 years); N = 8,197	USA	Cross-sectional	Townsend Material Deprivation Index (crowded occupied housing units, unemployed residents in the civilian labour force, tenant occupied housing units, occupied housing units without a vehicle available)	Number of alcohol outlets per square mile, distance to alcohol outlet from home address, number of alcohol outlets in a half mile radius around home address	**Individual:** age, sex, ethnicity, marital status, individual SEP index (income and education).
	**Perinatal and child health**						
Géneréux, 2008 [62]	**1.** Preterm birth (dichotomous); **2.** Low birth weight (dichotomous); **3.** Small for gestational age (dichotomous)	Life births; N = 99,819	Canada	Cross-sectional	Percentage of low-income families	Individual proximity to highway from home address (distance ≤200 m)	**Individual:** maternal age and education, infant´s sex, civil status, maternal country of birth, birth order, history of previous stillbirth, year of birth.
Ponce, 2005 [63]	Preterm birth (dichotomous)	Life births; N = 37,347	USA	Cross-sectional	Index (unemployed residents in the civilian labour force, households with public assistance income, families with income below the poverty line)	Distance-weighted traffic density based on individual distance to roadways from home address and annual average daily traffic counts	**Individual:** Maternal age, education, and ethnicity, payment for delivery, prenatal care, infant´s sex, parity, time since previous life birth, previous low birth weight or preterm infant, year of birth, live near highway, air pollutants. **Contextual:** season.
Williams, 2007 [60]	Birth weight in grams (continuous)	Life births; N = 13,559	USA	Cross-sectional	Percentage of residents below the poverty level	Average atmospheric concentration of sulphur dioxide, lead and fine particulates around infant´s home; number of hazardous waste sites in a 5 kilometre radius around infant´s home	**Individual:** maternal education and ethnicity, infant´s sex, previous infant delivery, previous infant >4,000 gram or <37 week, hypertension, oligohydramios, preeclampsia, previous non-live births, smoking, infants born from same pregnancy, other rare maternal risk factors.
Zeka, 2008 [61]	**1.** Birth weight in grams (continuous); **2.** Small for gestational age (dichotomous); **3.** Preterm birth (dichotomous)	Life births; N = 425,751	USA	Cross-sectional	Median annual household income	Cumulative average daily traffic; individual distance to major highways from home address; percentage of open space designed for recreation, conversation, water supply, and forestry	**Individual:** age of mother, maternal education, ethnicity, prenatal visits, gestational age, smoking during pregnancy, previous infant greater than 4,000 gram, previous preterm birth, chronic or gestational conditions of mother, year of birth.
Reading, 2008 [64]	**1.** Reported number of child accidents by mother; **2.** Reported number of medical attended child injuries	Children (0–5 years); N = 41,409	Britain	Longi-tudinal	Percentage of unemployed residents, percentage of social classes 4 and 5	Road density of all roads, road density of major roads, percentage of detached and semi-detached housing, percentage of terraced housing, percentage of purpose built flats, percentage of converted flats	**Individual:** child (age, sex, twin or triplet, ethnicity, physical activity, development, behavioural characteristics, motor functions, activity, risk avoidance, strength and difficulties, arguing with mother), mother (age, education, marital status, ethnicity, relationship status, partner moved out, lost partner, employment status, smoking status, alcohol and cannabis consume, depressive symptoms, significant life events, social support), partner (employment status, ethnicity, alcohol consumption), household (number and age of siblings, number of adults, lone parent, number of child caretakers, household income, financial difficulties, home owner, car), housing (rented, flat or room, garden, safety features), perceived neighbourhood environment (quality of neighbourhood, environmental problems, bus traffic, fear of crime, neighbourhood contacts), movement during data collection. **Contextual:** population aged 0–4 years, lone parents, movements, home owners, renters, overcrowded households, residents without a car, black population.

Abbreviations: SEP = Socioeconomic position; BMI = Body Mass Index; PM10 = quarterly measures of particulate matter at 10 μm or less

In a qualitative analysis independent and interactive effects of the built environment and neighbourhood SEP towards individual health outcomes were visualized in four tables grouped by similar health outcomes or health-related behaviours. All variables with a p-value <0.05 in the final multilevel model were reported as statistically significant. No quantitative assessment for risk of bias in individual studies was performed. However, in each study sample size, number of observations per neighbourhood and total number of considered neighbourhood clusters were checked, because simulation studies showed that small sample sizes in multilevel studies result in biased effect estimates [37–40]. The review was conducted in accordance to the PRISMA statement (S1 Table) [41].

## Results

**Fig 1:**
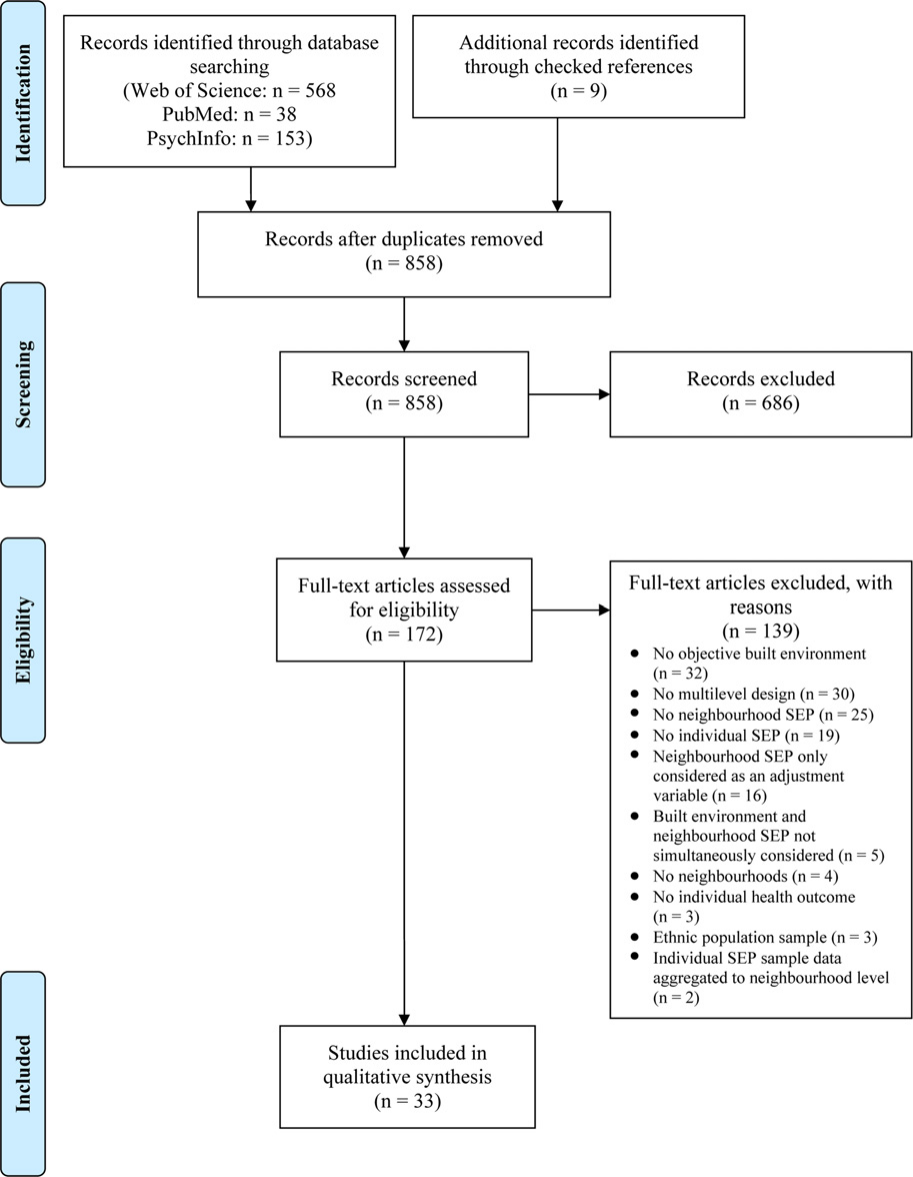
Flow diagram of study selection. The diagram describes the information flow containing number of identified records, included and excluded records, and the reasons why records were excluded. The diagram was adapted from the PRISMA statements [41].

After removing of duplicates 858 records were taken into account for abstract screening. 686 records were excluded based on abstracts and titles. There was a disagreement on 14 abstracts resulting in an agreement of 91.4% between the two independent reviewers. 172 records were included into full text analysis, and 24 of them met all inclusion criteria. Nine studies were additionally identified through the analysis of references from the 24 papers selected by full text analysis. These nine studies also underwent abstract screening and full text analysis. Finally, 33 studies were considered for qualitative analysis (Fig 1).

### Description of studies and sample size assessment

Except of one study, all had a cross-sectional study design and most of them were conducted in the United States (Table 2). Seven studies investigated exclusively outcomes measuring various forms of physical activity [42–48]. One of these seven studies analysed people 45 years or older [44] and one used data from adolescents aged 13–15 years [46]. The other five analysed an adult population sample.

Six studies examined exclusively measures indicating overweight or obesity either directly with the Body Mass Index (BMI) as a continuous variable or with BMI thresholds for overweight or obesity [49–54]. Two of them used data from older adults [49, 50].

Five studies analysed both measures of physical activity and overweight [55–59] including one study which considered additionally mental and physical quality of life, and depressive symptoms [59]. One of these studies used data from students aged 13–16 years [58].

Four studies investigated how neighbourhood context was associated with perinatal health outcomes [60–63] and one longitudinal study focused on child accidents and injuries in children aged 0–5 years [64]. Five studies analysed self-rated health or self-reported health problems [65–69], and two of them considered a population sample 55 years or older [67, 68]. One study focused exclusively on stress [70], one on smoking [71], one on heavy alcohol consumption [72] and two on objectively measured coronary artery calcification in adults aged 45–75 years [73, 74].

Regarding characteristics of neighbourhood SEP 16 studies calculated an index capturing various socioeconomic characteristics of the neighbourhood population [47, 49–52, 54–57, 65–72]. The others used single indicators of neighbourhood SEP, such as measures of income, education, poverty or unemployment.

The objective built environment was described with a variety of measures. Indices for walkability, land use mix and urbanity were calculated. Single land use types were also considered, such as retail, recreational areas, restaurants, fast food outlets, cultural and education institutions, or health and human services. Environmental pollution, such as from traffic or waste sites, was mainly investigated in studies focusing on perinatal health, mental health or self-rated health. Eleven studies calculated built environmental measures on the individual level [49, 51, 57, 60–63, 71–74], such as individual distances from residential addresses to shops or main roads.

There was a great heterogeneity concerning sample size both of individual observations and neighbourhood clusters. Sample size ranged from 637 to 425,752 individual observations and the number of considered neighbourhood clusters ranged from 24 up to 4,604 neighbourhoods. Unfortunately, only a minority of included studies gave detailed descriptive information about the number of observations per neighbourhood. Referring to simulation studies performed on sample sizes for multilevel models, most of the reviewed studies showed a sufficient size of neighbourhoods and individual observations [37–40]. However, due to missing information in many studies about the range of individual observations within neighbourhood clusters we could not assess whether these effect estimates could be biased.

### Associations between socioeconomic and built environments and physical activity

Two studies detected associations between neighbourhood SEP and physical activity independent from the built environment and individual factors (Table 3). In the first study a high neighbourhood income was negatively associated with walking for transport and positively with motorized transport [43], and in the second a high neighbourhood education was positively associated with various measures of walking [44]. One study found an interaction between neighbourhood SEP and sex [47]: The positive association between a high neighbourhood SEP and physical activity was mitigated for men.

**Table 3:** Associations between socioeconomic and built environments and physical activity.

**Reference**	**Outcomes** *	**Neighbourhood SEP** *				**Objective built environment** *																**Interactions**
		High SEP (index)	Low SEP (index)	High income	High edu-cation	Walk-ability	Urbanity index	Bike and walking path length	Conn-ectivity	Comp-actness index	Land use mix	Traffic and air pollution	Parks and green space	Sport facilities	Cultural/ edu-cational institu-tions	Restau-rants	Fast food outlets	Retail	Health and human services	Dis-tance to parks	Dis-tance to subway	
De Meester, 2012 [46]	Average activity level			M		⇆																⊕ only in low income neighbourhoods
	MVPA			M		⇆																⊕ only in low income neighbourhoods
	Walking, cycling, and sport			n.s.		n.s.																No significant interactions detected
	Active transport to and from school			n.s.		n.s.																No significant interactions detected
Owen, 2007 [48]	Walking for transport (weekly minutes)			n.s.		⇵																⊕ only for people who choose place due to service access
	Walking for transport (weekly frequency)			n.s.		⇵																⊕ only for people with 12 or more years of education
	Walking for recreation			n.s.		n.s.																No significant interactions detected
Prince, 2012 [55]	Leisure time physical activity		n.s.					n.s.					⇵	n.s.		n.s.	n.s.	n.s.				⊕ only for women
Prince, 2011 [56]	Overall physical activity		n.s.					n.s.					⇵^1)^	n.s.		⇵^2)^	n.s.	⇵^3)^				1) ⊖ only for men; 2) ⊕ only for women; 3) ⊕ only for men
Riva, 2009 [44]	Walking per week for any motive				⊕		n.s.															Not reported
	Utilitarian walking per week				⊕		⊕															Not reported
	Recreational walking per week				⊕		⊖															Not reported
Sallis, 2009 [59]	MVPA			n.s.		⊕																No significant interactions detected
	Walking for leisure			n.s.		n.s.																No significant interactions detected
	Walking for transportation			n.s.		⊕																No significant interactions detected
Scott, 2009 [57]	Utilitarian walking	n.s.							n.s.				n.s.					⇵				⊕ only for non-Hispanic whites
	Recreational walking	n.s.							⇵				n.s.					n.s.				⊖ only for African Americans (street connectivity (alpha index)); ⊖ stronger for non-Hispanic whites (block length)
Slater, 2010 [58]	Vigorous exercise			n.s.						n.s.		n.s.		n.s.								No significant interactions detected
	Sports participation			n.s.						⊖		n.s.		⊕								No significant interactions detected
	Physical activity participation			n.s.						n.s.		n.s.		n.s.								No significant interactions detected
Sundquist, 2011 [45]	MVPA			n.s.		⊕																No significant interactions detected
	Walking for active transportation			n.s.		⊕																No significant interactions detected
	Walking for leisure			n.s.		⊕																No significant interactions detected
Van Dyck, 2010 [43]	MVPA			n.s.		⊕																No significant interactions detected
	Walking for transport			⊖		⊕																No significant interactions detected
	Walking for recreation			n.s.		⊕																No significant interactions detected
	Cycling for transport			n.s.		⊕																No significant interactions detected
	Motorized transport			⊕		⊖																No significant interactions detected
Van Dyck, 2010 [42]	Sitting time			n.s.		⊕																No significant interactions detected
	Sedentary time			n.s.		⊕																No significant interactions detected
Wen, 2009 [47]	Weekly workout/exercise	⇵									n.s.				n.s.	⊕			n.s.	n.s.	n.s.	⊕ mitigated for men
	Regular exercise past year	⇵									n.s.				n.s.	⊕			n.s.	n.s.	n.s.	⊕ mitigated for men

* a detailed description of variables is given in table 2

Abbreviations: SEP = Socioeconomic position; MVPA = Moderate-to-vigorous physical activity; BMI = Body Mass Index; ⇆ = Within-level interaction (interaction is specified in the interaction column); ⇵ = Cross-level interaction (interaction is specified in the interaction column); ⊕ = Significant positive association; ⊖ = Significant negative association; n.s. = Not significant; M = Variable considered as a moderator via stratification or interaction term

Seven studies detected associations between the built environment and physical activity measures independent from neighbourhood SEP and individual factors. Three found a positive association between a walkability index and walking behaviours and physical activity [43, 45, 59]. Moreover, a walkability index was inversely associated with motorized transport [43]. One study detected an unexpected positive association between a walkability index and self-reported sitting behaviour and objectively measured sedentary time [42]. Urbanity was positively associated with utilitarian walking and negatively with recreational walking [44]. One study detected an independent positive association between number of restaurants and bars and regular exercise [47]. A further study analysing physical activity in students found a negative association between a calculated compactness index and sport participation and a positive association between number of sport facilities and sport participation [58].

Most studies considering single land use types, such as retail or recreational areas, found no associations. In five studies interactions were detected. Studies showed that associations between recreational land use, retail or availability of restaurants varied by sex or ethnicity [55–57]. One study showed a positive association between park areas and leisure time physical activity only for women [55]. An inverse association between green space and overall physical activity was observed only for men [56]. The same study showed a positive association between number of restaurants and overall physical activity only for women and a positive association between number of convenience stores and overall physical activity only for men. A third study demonstrated a positive association between number of markets and utilitarian walking only for non-Hispanic whites, a negative association between street connectivity and recreational walking only for African Americans, and a negative association between block length and recreational walking was slightly stronger for non-Hispanic whites [57].

One study detected two interactions: One between a walkability index and individual reported reasons why people choose their neighbourhood and another between the walkability index and education. There was a positive association between a walkability index and walking for transport only for people who choose their neighbourhood because of a good perceived neighbourhood environment (closeness to job, school, shops, services or good perceived walkability) and only for people with 12 or more years of education [48]. In a second study neighbourhood income moderated the association between walkability and physical activity. A positive association between walkability and two outcomes of average activity level and moderate-to-vigorous physical activity was only significant in low income neighbourhoods [46].

### Associations between socioeconomic and built environments and overweight

Eight of eleven studies showed significant associations between indicators of neighbourhood SEP and BMI, overweight or obesity independent from individual and built environmental factors (Table 4). Three studies found a negative association between a high neighbourhood SEP and BMI, overweight or obesity [49, 50, 52]. Two found a positive association between a low neighbourhood SEP and BMI [51, 54]. A high neighbourhood income was negatively associated with BMI and obesity [58], a low neighbourhood income was positively associated with BMI and obesity [59], and a low neighbourhood education was positively associated with BMI [53].

**Table 4:** Associations between socioeconomic and built environments and measures of overweight.

**Reference**	**Outcomes** *	**Neighbourhood SEP** *					**Objective built environment** *													**Interactions**
		High SEP (index)	Low SEP (index)	High income	Low income	Low edu-cation	Low walka-bility	Bike/ walking path length	Connec-tivity	Compa-ctness index	Traffic and air pollution	Dwelling density	Density index	Parks and green space	Sport facilities	Dis-tance to parks	Res-taurants	Fast food outlets	Retail	
Inagagami, 2009 [54]	BMI		⊕														⊕	⇵		⊕ mitigated for car owners
Moore, 2013 [49]	BMI	⊖													⊖				n.s.	Not reported
Ross, 2007 [53]	BMI			n.s.		⊕						n.s.								No significant interactions detected by sex
Scott, 2009 [57]	BMI	⇵							⇵					**⇆**					n.s.	⊖ only for non-Hispanic whites (alpha index)
Wang, 2007 [51]	BMI		⊕															n.s.	^1)^ **⇆** ^2)^⇵	1) ⊖ only for women (individual proximity to ethnic markets and supermarket); 2) ⊕ only for women (density of grocery stores)
Slater, 2010 [58]	BMI			⊖						⊖	n.s.			n.s.						No significant interactions detected
	Obesity			⊖						⊖	n.s.			n.s.						No significant interactions detected
Sallis, 2009 [59]	BMI				⊕		n.s.													No significant interactions detected
	Overweight or obesity				n.s.		⊕													No significant interactions detected
	Obesity				⊕		n.s.													No significant interactions detected
Grafova, 2008 [50]	Obesity	⊖	n.s.						n.s.		⇵		n.s.							⊖ only for women (air pollution)
	Overweight or obesity	⊖	n.s.						n.s.		n.s.		n.s.							No significant interactions detected
Prince, 2012 [55]	Overweight or obesity		n.s.					n.s.						⇵	n.s.		n.s.	⇵	⇵	⊕ only for women
Prince, 2011 [56]	Overweight or obesity		⇵^1)^					n.s.						⇵^2)^	⇵^3)^		⊖	n.s.	⇵^4)^	1) ⊖ only for men; 2) ⊕ for men and ⊖ for women; 3) ⊕ only for women (summer outdoor facilities); 4) ⊕ only for women (specialty stores)
Wen, 2012 [52]	Obesity	⊖							⊖							⊕				No significant interactions detected by sex

* a detailed description of variables is given in table 2

Abbreviations: SEP = Socioeconomic position; MVPA = Moderate-to-vigorous physical activity; BMI = Body Mass Index; ⇆ = Within-level interaction (interaction is specified in the interaction column); ⇵ = Cross-level interaction (interaction is specified in the interaction column); ⊕ = Significant positive association; ⊖ = Significant negative association; n.s. = Not significant; M = Variable considered as a moderator via stratification or interaction term

Two studies on overweight showed interactions between neighbourhood SEP and individual characteristics. One study found out that an unexpected inverse association between a low neighbourhood SEP and overweight or obesity was only significant for men [56]. A further study detected a negative association between a high neighbourhood SEP and BMI only for non-Hispanic whites [57].

Seven studies detected significant associations between built environmental factors and measures of overweight independent from neighbourhood SEP and individual characteristics. Two studies detected an independent inverse association between measures of street connectivity and BMI or obesity [52, 58] and one of them showed a positive association between distances to parks and obesity [52]. Low walkability was positively associated with overweight or obesity in one study [59]. There was a negative association between density of sport facilities in a one mile radius around home address and BMI [49]. A further study found an unexpected negative association between number of restaurants and overweight or obesity [56]. A further study showed a positive association between number of restaurants and BMI, however, the total number of restaurants included also fast food outlets [54].

In six studies interactions were shown. A negative association between street connectivity, number of parks in a one mile radius around the home address and BMI was only significant for non-Hispanic whites [57] and an inverse association between air pollution and obesity was only significant for women [50]. One study showed a significant positive association between number of convenience stores, fast food restaurants, park areas and overweight or obesity only for women [55]. A further study detected that a positive association between fast food outlets and BMI was mitigated for car owners [54].

There was a negative association between proximity to ethnic markets and supermarkets from home address and BMI only for women and, on the other hand, a positive association between neighbourhood density of grocery stores and BMI also only for women [51]. A further study detected a significant positive association between number of specialty stores and overweight or obesity only for women, too. The same study found an inverse association between green space and overweight or obesity for women and a positive association for men, however. Moreover, a significant positive association between summer outdoor facilities and the two outcomes of overweight and obesity was also only detected for women [56].

### Associations between socioeconomic and built environments and health outcomes and health-related behaviours

Most studies detected significant associations between neighbourhood SEP and health outcomes or health-related behaviours (Table 5). Six studies found significant associations between neighbourhood SEP and individual health outcomes or health-related behaviours independent from the built environment and individual characteristics. A high neighbourhood income was positively associated with physical quality of life [59]. One study found a positive association between a high neighbourhood SEP and heavy alcohol consumption [72]. A further study detected a positive association between neighbourhood unemployment and artery calcification [74]. There was an inverse association between a low neighbourhood SEP and a health score derived from self-reported health problems. Higher values of the score indicated better health [66]. Neighbourhood unemployment was positively associated with bad self-rated health [65]. One study which analysed disability in people aged 55 or older detected a negative association between a high neighbourhood SEP and minor reported body limitations [68].

**Table 5:** Associations between socioeconomic and built environments and health outcomes and health-related behaviours.

**Reference**	**Outcomes** *	**Neighbourhood SEP** *				**Objective built environment** *													**Interactions**
		Low SEP (index)	High SEP (index)	High income	High unem-ploy-ment	Walk-ability	Street connec-tivity	Traffic and air pollution	Waste sites	Low public re-creation score	Low bank/ building society score	Low environ-ment score	Low health service score	Density index	Distance to and numbers of stores	Store density	Alcohol outlet density	Low food score	
Cummins, 2005 [65]	Self-rated health				⊕					n.s.	n.s.	⇵	n.s.					n.s.	⊕ stronger for non-working study participants
Stafford, 2005 [69]	Self-rated health				⇵^1)^					n.s.	⇵^1)^	⇵^1)^	⇵^1)^					⇵^2)^	1) ⊕ only for women; 2) ⊕ only for men
Freedman, 2011 [67]	Heart Problems	⇵	n.s.				n.s.	n.s.						n.s.					⊕ only for women
	Blood Pressure, Stroke, Cancer Diabetes, Arthritis	n.s.	n.s.				n.s.	n.s.						n.s.					No significant interactions detected
Freedman, 2008 [68]	Body limitations	n.s.	⊖				n.s.	n.s.						n.s.					No significant interactions detected
	Daily activity limitations	⇵	n.s.				n.s.	n.s.						n.s.					⊕ only for men
	Instrumental activity limitations	n.s.	n.s.				n.s.	n.s.						n.s.					No significant interactions detected
Matthews, 2010 [66]	Health score	⊖	M ⇵^1)^					⊖	M ⇵^2)^										1) ⊖ for high individual stress: mitigated in more affluent neighbourhoods; 2) ⊖ for high individual stress: stronger in areas with residual waste operations
Sallis, 2009 [59]	Physical quality of life			⊕		n.s.													No significant interactions detected
	Mental quality of life			n.s.		⊖													No significant interactions detected
	Depressive symptoms			n.s.		⊕													No significant interactions detected
Yang, 2010 [70]	Day-to-day stress	n.s.						⊕	⊕										Not reported
Dragano, 2009 [73]	Artery calcification				⇵			M											⊕ only for women with a distance to major road >100 m and ⊕ only for men with a distance to major road <100 m
Dragano, 2009 [74]	Artery calcification				⊕			**⇆**											⊕ only for men with a distance to major roads ≤50 m
Chuang, 2005 [71]	Smoking		⇵^1)^												⇵^2); 3)^	⇵^4)^ **⇆** ^5)^			1) ⊖ stronger for high SEP individuals; 2) ⊖ stronger in high SEP neighbourhoods (individual distance to stores); 3) ⊕ stronger in high SEP neighbourhoods (individual number of stores around home address); 4) ⊕ mitigated for high SEP individuals; 5) ⊕ stronger in high SEP neighbourhoods
Pollack, 2005 [72]	Alcohol consumption		⊕														n.s.		No significant interactions detected

* a detailed description of variables is given in table 2

Abbreviations: SEP = Socioeconomic position; MVPA = Moderate-to-vigorous physical activity; BMI = Body Mass Index; ⇆ = Within-level interaction (interaction is specified in the interaction column); ⇵ = Cross-level interaction (interaction is specified in the interaction column); ⊕ = Significant positive association; ⊖ = Significant negative association; n.s. = Not significant; M = Variable considered as a moderator via stratification or interaction term

Three studies detected associations between built environmental factors and health outcomes independent from neighbourhood SEP and individual factors. A walkability index was inversely associated with mental quality of life and positively with more depressive symptoms [59]. One study found a negative association between traffic and a health score calculated from self-reported health problems. Higher values of the score indicated better health [66]. In another study waste sites and traffic were positively associated with reported day-to-day stress [70].

In eight studies factors of neighbourhood SEP or the built environment interacted with individual characteristics. There was a positive association between a low neighbourhood SEP and reported heart problems only for women aged 55 years or older [67]. One study detected that an inverse association between a high neighbourhood SEP and number of smoked cigarettes per day was stronger for residents with a higher individual SEP [71]. Neighbourhood unemployment interacted both with sex and traffic in a further study. For men there was a positive association between neighbourhood unemployment and artery calcification with an individual distance to the next major road <100 meter from their home address. Unexpectedly, a positive association between neighbourhood unemployment and artery calcification was significant for women with a distance to the next major road >100 meter [73]. Another study found a positive association between neighbourhood unemployment and bad self-rated health only for women [69]. One study considered neighbourhood SEP as a moderator on the association between individual reported stress and a calculated health score from self-reported health problems. Higher values of the score indicated better health. The negative association between higher reported stress and a higher health score was mitigated in areas with a high neighbourhood SEP and stronger in areas with present residual waste operations [66]. In a further study, a positive association between a low neighbourhood SEP and self-reported limitations in daily activities from people aged 55 or older was only significant for men [68].

In the only identified study on smoking, the positive association between convenience store density per square mile and number of smoked cigarettes per day was mitigated for individuals with a higher SEP and, in contrast, was stronger in neighbourhoods with a high SEP. A positive association between number of convenience stores in a one mile radius around home address and number of smoked cigarettes was also stronger in neighbourhoods with a high SEP. Furthermore, a negative association between distance to convenience stores from home address and number of smoked cigarettes per day was stronger in neighbourhoods with a high SEP [71].

One study found a positive association between distance to a major road ≤50 m from home address and artery calcification only for men [74]. One study on self-rated health showed a positive significant association between a lower food score and bad self-rated health only for men. Lower values of three other scores (bank/building society score, physical environment score, health service score) were significantly positively associated with bad self-rated health only for women [69]. A further study exploring the same built environmental variables found out that a low physical environment score was positively associated with bad self-rated health and was stronger for non-working study participants [65].

### Associations between socioeconomic and built environments and perinatal outcomes and child health

Most studies on perinatal health found interactive associations (Table 6). Only one study showed that a high neighbourhood income was independently positively associated with birth weight and negatively with small for gestational age [61]. A further study on child accidents and injuries did not find an association either of neighbourhood unemployment nor the built environment [64].

**Table 6:** Associations between socioeconomic and built environments and perinatal outcomes and child health.

**Reference**	**Outcomes** *	**Neighbourhood SEP** *					**Objective built environment** *								**Interactions**
		SEP index	High income	Low income	Unemploy-ment	Poverty	Air pollution	Traffic	Proximity to highways	Distance to highways	Road density	Waste sites	Open space	Building character-istics	
Géneréux, 2008 [62]	Preterm birth			M					**⇆**⇵						⊕ only in neighbourhoods with a high income and for mothers with a university education
	Low birthweight			M					**⇆**⇵						⊕ only in neighbourhoods with a high income and for mothers with a university education
	Small for gestational age			M					⇵						⊕ only in neighbourhoods with a high income
Ponce, 2005 [63]	Preterm birth	M						⇵							⊕ only in low SEP neighbourhoods during winter
Williams, 2007 [60]	Birth weight					⇵	⊖					n.s.			⊖ stronger for individuals with rare maternal risk factors
Zeka, 2008 [61]	Birth weight		⊕					n.s.		**⇆**			⇵		⊕ only for mothers with a high education
	Small for gestational age		⊖					n.s.		n.s.			n.s.		No significant interactions detected
	Preterm birth		n.s.					n.s.		n.s.			n.s.		No significant interactions detected
Reading, 2008 [64]	Child accidents and injuries				n.s.						n.s.			n.s.	not analysed

* a detailed description of variables is given in table 2

Abbreviations: SEP = Socioeconomic position; MVPA = Moderate-to-vigorous physical activity; BMI = Body Mass Index; ⇆ = Within-level interaction (interaction is specified in the interaction column); ⇵ = Cross-level interaction (interaction is specified in the interaction column); ⊕ = Significant positive association; ⊖ = Significant negative association; n.s. = Not significant; M = Variable considered as a moderator via stratification or interaction term

One study gave an interaction between neighbourhood SEP and maternal risk factors. The overall inverse association between neighbourhood poverty and lower birth weight was stronger for individuals with rare maternal risk factors [60].

Regarding the built environment mostly traffic-related measures were analysed. Only one study found an independent negative association between air pollutants and birth weight [60]. All other built environmental factors interacted with neighbourhood SEP or individual factors or were not significant. One study detected a positive association between distance to highways from home address, percentage of open space and birth weight only for mothers with a high education [61]. In one study a positive association between proximity to highways and three outcomes of preterm birth, low birth weight, and small for gestational age was only significant in neighbourhoods with a high neighbourhood income. Moreover, the positive association between proximity to highways from home address and the two outcomes of preterm birth and low birth weight were only significant for mothers with a university education [62]. However, in a further study a positive association between distance weighted traffic density and preterm birth was only significant in neighbourhoods with a low SEP index in winter time [63].

## Discussion

This systematic review identified and qualitatively analysed studies applying multilevel modelling which simultaneously considered characteristics of neighbourhood SEP and the objective built environment and analysed their effects on individual health outcomes or health-related behaviours.

Sixteen studies found associations between neighbourhood SEP and individual health independent from built environmental and individual characteristics. Fourteen studies showed associations between built environmental characteristics and individual health independent from neighbourhood SEP and individual characteristics. In seven studies simultaneous independent associations of neighbourhood SEP and the objective built environment were identified. Twenty-one studies showed cross-level or within-level interactions either between neighbourhood SEP and the built environment, or between neighbourhood SEP and individual characteristics, or between the built environment and individual characteristics.

Although we grouped our studies by similar health outcomes, a systematic assessment to what extend neighbourhood SEP and the built environment influenced individual health and health-related behaviour independently and dependently from each other or were modified by individual characteristics was difficult. The most frequently analysed outcomes were measures of physical activity and overweight. A lower neighbourhood SEP was mostly associated with higher BMI, overweight, obesity, bad self-rated health or artery calcification independent from built environmental and individual factors [51, 53, 54, 59, 65, 66, 74]. However, most studies analysing measures of physical activity did not find associations between neighbourhood SEP and measures of physical activity. Objective built environmental metrics indicating higher walkability were often associated with measures of higher individual physical activity independent from neighbourhood SEP and individual factors [43, 45, 59].

This review showed that interactions play an important role. Individual characteristics, such as sex, ethnicity or individual SEP, often modified associations between neighbourhood SEP or the objective built environment and individual health. However, it became not clear how neighbourhood SEP and built environmental characteristics interacted with sex. There were characteristics of the built environment and neighbourhood SEP from which only women´s or men´s health benefited or suffered [50, 51, 55, 56, 67–69, 73, 74]. No systematic findings which specific factors of neighbourhood SEP or the built environment are more harmful to men´s or women´s health could be detected.

Various moderating associations of neighbourhood SEP on associations between the built environment and health were identified. Some studies observed an impact of a health promoting built environment only in neighbourhoods with a low SEP [46]. On the other hand there were studies demonstrating that associations between factors of a higher built environmental burden and poor health or negative health behaviours were only significant or stronger in neighbourhoods with a high SEP [62, 71]. In contrast, one study found out that only individuals living in a low SEP neighbourhoods were affected by higher built environmental burdens [63].

A variety of measures both of socioeconomic and built environments were studied which may partly explain mixed results. Especially concerning metrics and definitions of built environmental variables, there was a great heterogeneity. The majority of all studies calculated weighted numbers of various facilities, such as stores, sport and recreational facilities, parks, or restaurants. There was a great variety concerning the weights that were used. The most used weights were: fixed number of residents, number of neighbourhood residents, neighbourhood size or square kilometre, distance based buffer around each individual´s home address, or the centroid of the neighbourhood. Moreover, many studies calculated indices, mostly derived from factor analysis. The number and kind of built environmental variables contained in these scores was too heterogeneous for drawing comparisons. The only comparable index across studies was the walkability index. A minority of studies calculated distance based measures, such as to main roads, stores or parks. Distances were calculated either from individual home addresses or from neighbourhood centroids. The limited comparability across studies is consistent with previous systematic reviews, which focused either on a specific health outcome or exclusively on one neighbourhood environmental dimension [18, 21, 26, 28].

A further explanation for inconsistent results could be that built environments and socioeconomic neighbourhood structures vary across countries and continents. Besides that, these variations can be shaped by country specific social and housing policies on the neighbourhood level.

Studies were included which considered at least one individual socioeconomic factor, one factor of neighbourhood SEP and one of the objective built environment. However, apart from sex and age, studies varied by the number of included individual and contextual variables that might explain mixed results, too. Individual data on health behaviours, such as smoking or nutrition, and family status (e.g. marital status) were in some studies additionally considered. Many studies included also factors of the social environment, such as crime or characteristics of social capital in the neighbourhood. Individual and contextual characteristics may mediate associations on the pathway between neighbourhood SEP and individual health or between built environmental factors and individual health. The study by Reading et al. is an example where individual factors completely mediated the association between neighbourhood context and child accidents [64].

### Limitations

A first limitation is that our qualitative analysis only visualized significance or non-significance and direction of associations or interactions and did not make any comparisons on strength of the associations. The operationalization of variables was too heterogeneous across studies to perform meaningful quantitative comparisons. A second limitation is that our search code was mainly based on title and abstract screening. Besides that the Medical Subject Headings used in the PubMed database may not correspond to selected keywords by authors. Therefore, our search strategy was maybe not sensitive enough and could not identify all relevant studies. To reduce this limitation, we checked all references of included studies. We assumed that there were no relevant studies in grey literature. Therefore, we did not perform a separate search in sources of grey literature. Our assumption was sustained by the fact that we could not identify relevant grey literature which was cited in included studies.

### Strengths

The main strength of this review is that we exclusively focused on studies which considered both characteristics of neighbourhood SEP and the objective built environment simultaneously in multilevel models with the additional consideration of individual factors. We were able to analyse how these two neighbourhood dimensions were interrelated and interacted with individual variables. This systematic interaction analysis on both the neighbourhood and the individual level revealed new insight which role these dimensions play in epidemiological neighbourhood research and identified where further research is needed. A further strength is that the systematic search was not restricted to specific health outcomes or age and population groups. As a result, we could identify for which health outcomes, health-related behaviours or population groups evidence is lacking or results are most inconsistent.

### Recommendations for future research

Based on our results we suggest the following recommendations for future research: Firstly, the consideration of more than one environmental neighbourhood dimension is important for generating more evidence on how socioeconomic, built and social neighbourhood characteristics are associated with individual health. It offers the possibility to analyse mediating and interacting pathways. There is still a lack of knowledge to what extent the built environment mediates effects of neighbourhood SEP on individual health. Being aware of potential reciprocal relationships between neighbourhood SEP and the built environment provides a better basis analysing interactions with individual characteristics, such as sex, individual SEP or health behaviours. Increasing knowledge about the health impact of the built environment will contribute to the reconnection of urban planning and public health. There is an upcoming call in public health sciences that for a sustainable healthy city development there is a need for updating and refreshing the connection between urban planning and public health [24, 75]. Moreover, conceptual models from the scientific field of risk assessment with a specific focus on different forms of vulnerability and cumulative environmental exposures on the individual and neighbourhood level may provide a good basis for identifying synergies between vulnerability analysis in epidemiology and cumulative risk assessment [76, 77].

Secondly, there is a need to adhere to guidelines on how results from multilevel modelling should be reported. One key feature of multilevel models is that they are able to sort out variance components both on the neighbourhood and individual level which provide important information how individual health varies between and within neighbourhoods and how much of these variance can be explained by contextual factors. However, to give a systematic overview about variance components and to draw conclusions on how much of the built and socioeconomic environment contributes to health disparities was impossible in this review, because some studies reported measures on variance components and some not. Already existing glossaries and tutorials about multilevel modelling, which support a better reporting of multilevel results should receive more attention [12, 78, 79].

Thirdly, in some studies it was not clear what kind of cross-level or within-level interactions was analysed. Therefore, we encourage researchers to systematically report if and which cross-level and within-level interactions were analysed regardless of their statistical significance. Moreover, most studies did not provide descriptive statistics about sample sizes of individual observations per neighbourhood cluster which is important for assessing potential bias of effect or variance estimates. Publishing such statistical information would make quantitative comparisons of multilevel models across studies easier.

Fourthly, our review revealed a great heterogeneity of metrics and definitions of variables describing the built environment. The only and most consistent index across studies was the walkability index. More of such standardized indices measuring the built environment would increase comparability across studies. The application of GIS which are increasingly used in public health research can facilitate this development especially when distance based measures are developed.

Fifthly, all of our identified studies, except of one, were cross-sectional and therefore results should be interpreted with caution. There is a need of conducting studies with a longitudinal design to prevent the problem of reverse causation.

Finally, more studies considering both environmental dimensions are needed which focus on children, because they are more vulnerable to environmental burdens than other population groups [80]. Moreover, there is a lack of studies analysing how neighbourhood SEP and built environmental factors influence mental health outcomes, such as depression, or health-related risk behaviours, such as smoking or alcohol consumption.

## Conclusions

This systematic review showed that a simultaneous consideration of neighbourhood SEP, built environmental characteristics and individual factors is important for analysing pathways how neighbourhood context influence individual health outcomes and health-related behaviours. There is a need for comparable studies considering multiple neighbourhood dimensions and analysing interactive and mediating processes both between contextual factors and individual characteristic and between contextual factors itself because our review identified mixed results. For an integrated analysis of both aggregated neighbourhood SEP and built environmental factors a multilevel modelling approach is appropriate because it allows to consider individual factors concurrently. This study design can generate more evidence to what extent the built environment mediates associations between neighbourhood SEP and health, and how individual characteristics, such as sex or individual SEP, act as effect modifiers in order to identify vulnerable neighbourhoods and population groups.

## Supporting Information

S1 Table PRISMA 2009 checklist. (PDF)

S1 Text Search term for PubMed, PsychInfo, and Web of Science. (PDF)
