# Correction: Interactive and Independent Associations between the Socioeconomic and Objective Built Environment on the Neighbourhood Level and Individual Health: A Systematic Review of Multilevel Studies

**DOI:** 10.1371/journal.pone.0189552

**Published:** 2017-12-07

**Authors:** The PLOS ONE Staff

In Fig 1, the number of records identified in the PubMed database is incorrect. Please see the correct Fig 1 here.

**Fig 1:**
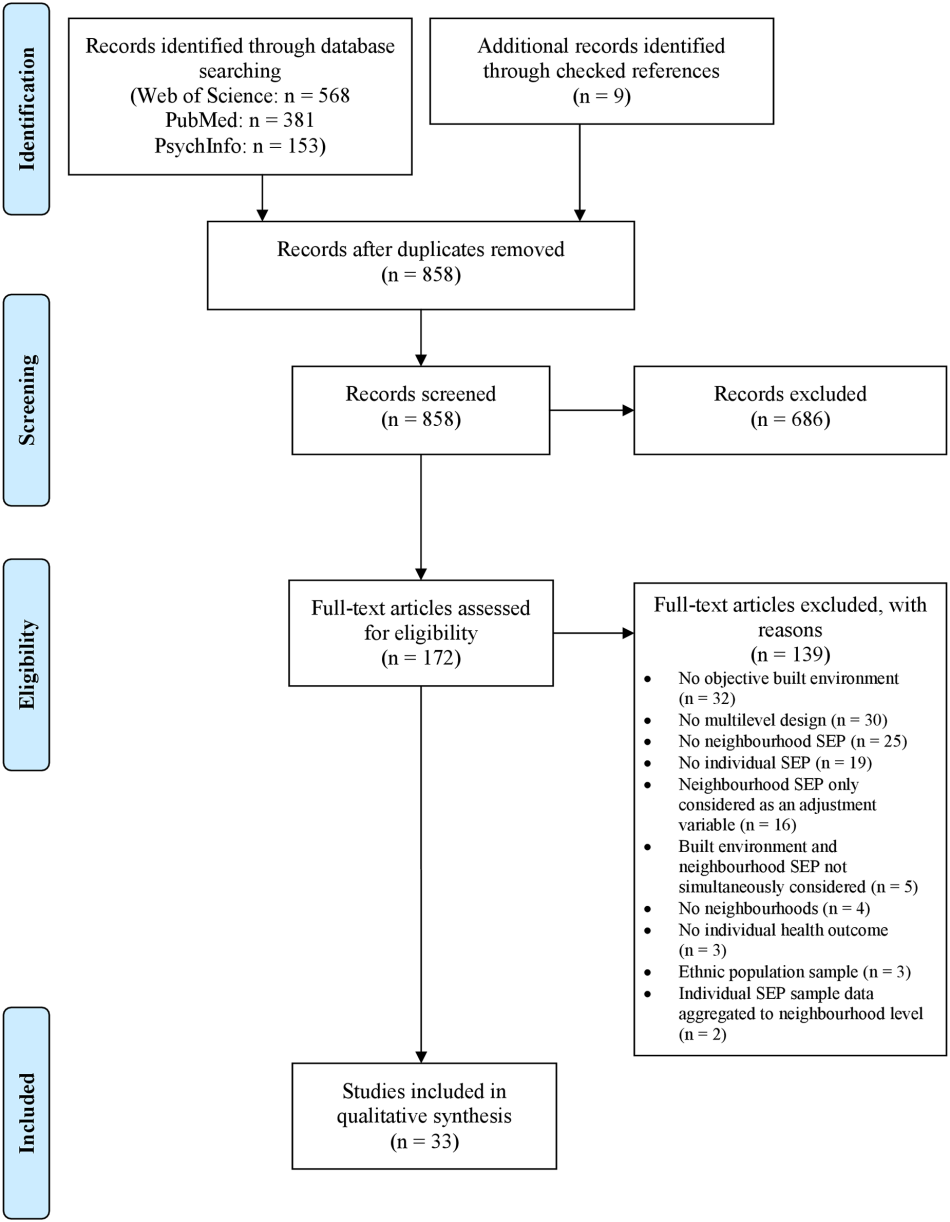
Flow diagram of study selection. The diagram describes the information flow containing number of identified records, included and excluded records, and the reasons why records were excluded. The diagram was adapted from the PRISMA statements [41].

Several studies are missing from Table 2. The publisher apologizes for the error. Please see the complete, correct Table 2 here.

**Table 2:** Description of studies.

Reference	Health outcomes	Sample	Country	Design	Neighbourhood factors		Individual and further contextual factors considered in multilevel analysis
					Neighbourhood SEP	Objective built environment	
	**Physical activity, overweight, quality of life, and depressive symptoms**						
De Meester, 2012 [46]	- Average activity level in counts per minute and moderate-to-vigorous physical activity in mean minutes per day assessed with accelerometer (continuous) - Reported walking, cycling, and sport during leisure time, and active transport to and from school in minutes per day (continuous)	Adole-scents (13–15 years) N = 637	Belgium	Cross-sectional	Median annual household income	Walkability index (residential density, intersection density, land use mix)	Individual: educational attainment and employment status of parents
Owen, 2007 [48]	- Reported walking for transport in minutes per week and number of days past week (continuous) - Reported walking for recreation in minutes per week and number of days past week (continuous)	Adults (20–65 years) N = 2,650	Australia	Cross-sectional	Median annual household income	Walkability index (dwelling density, street connectivity, land use mix, net retail area)	Individual: age, sex, education, annual household income, children in household, reported reasons for neighbourhood self-selection
Prince, 2012 [55]	- Reported physical activity (dichotomous): inactive and moderately physical activity vs. high physical activity - Under-/normal weight vs. overweight/obesity (dichotomous)	Adults (≥18 years) N = 4,727	Canada	Cross-sectional	Index (households below the low-income cut-off, average household income, unemployed residents, residents with less than a high school education, single-parent families)	Number of winter indoor/outdoor facilities and summer outdoor facilities per 1,000 residents; green space and park area (km^2^); bike/walking path length (km); number of grocery stores, fast food outlets, convenience stores, restaurants, and speciality food stores per 1,000 residents	- Individual: age, sex, education, household income, smoking status, season of data collection, community belonging - Contextual: councillor voting rates, crime, season
Prince, 2011 [56]	- Reported physical activity (dichotomous): inactive and moderately physical activity vs. high physical activity - Under-/normal weight vs. overweight/obesity (dichotomous)	Adults (≥18 years) N = 3,514	Canada	Cross-sectional	Index (households below the low-income cut-off, average household income, unemployed residents, residents with less than a high school education, single-parent families)	Number of winter indoor/outdoor facilities and summer outdoor facilities per 1,000 residents; green space and park area (km^2^); bike/walking path length (km); number of grocery stores, fast food outlets, convenience stores, restaurants, and speciality food stores per 1,000 residents	- Individual: age, sex, education, household income, smoking status, community belonging - Contextual: councillor voting rates, crime, season
Riva, 2009 [44]	Reported number of 10-minute episodes walking in the last seven days (continuous): walking for any motive, utilitarian walking, recreational walking	Adults (≥45 years) N = 2,923	Canada	Cross-sectional	Percentage of residents with a university education	Urbanity index (population density, land use mix, and accessibility to services)	Individual: age, sex, education
Sallis, 2009 [59]	- Moderate-to-vigorous physical activity in minutes per day assessed with accelerometer (continuous) - Reported walking for leisure and transportation (continuous): minutes per week - BMI (continuous) - Overweight or obesity (dichotomous) - Obesity (dichotomous): - Reported physical quality of life (continuous) - Reported mental quality of life (continuous) —Reported depressive symptoms (continuous)	Adults (20–65 years) N = 2,199	USA	Cross-sectional	Median annual household income	Walkability index (residential density, retail floor area ratio, mixed land use, intersection density)	Individual: age, sex, education, ethnicity, number of adults and motor vehicles in household, marital status, number of people in household, number of years living at current address, reported reasons for moving to neighbourhood
Scott, 2009 [57]	- Reported number of days per week walking to school, to work, to a store, to the bus, to do an errand, or to a neighbour’s house (continuous) - Reported number of days per week walking outdoors just for exercise or pleasure (continuous) -BMI (continuous)	Adults (≥18 years) N = 1,815	USA	Cross-sectional	Index (adults older than 25 with less than a high school education, male unemployment, households with income below the poverty line, households receiving public assistance, households with children headed only by a female, median household income)	Number of parks and markets in a one mile radius around home address, street connectivity, median block length, street density	Individual: age, sex, ethnicity, income, access to car in the household, perceived neighbourhood safety, utilitarian and recreational walking considered as an adjustment variables in the final BMI model
Slater, 2010 [58]	- Reported vigorous exercise (dichotomous) - Reported sports participation (dichotomous) - Reported physical activity participation (dichotomous) - BMI (continuous) - Obesity	Students (13–16 years) N = 10,620–36,929	USA	Cross-sectional	Median annual household income	Number of physical activity outlets per 10,000 residents, ratio of higher road classes to all other roads, compactness index (residential density and street connectivity)	- Individual: sex, grade, ethnicity, parental education, student income, students work, mother´s work status, private or public school, region, year of data collection, perceived environment (physical disorder scale, physical activity setting scale, perceived neighbourhood safety - Contextual: community observations by trained field teams (advertising, recreational space, social interactions, events, safety, general upkeep of the area),
Sundquist, 2011 [45]	- Moderate-to-vigorous physical activity in minutes per day assessed with accelerometer (continuous) - Reported walking for active transportation and leisure (continuous and dichotomous)	Adults (20–66 years) N = 2,269	Sweden	Cross-sectional	Median family income	Walkability index (residential density, street connectivity, land use mix)	Individual: age, sex, family income, marital status
Van Dyck, 2010 [42]	- Sedentary time assessed with accelerometer: percentage of wearing time below 100 counts per minute (continuous) - Reported sitting time in the past 7 days in minutes per day (continuous)	Adults (20–65 years) N = 1,200	Belgium	Cross-sectional	Median annual household income	Walkability index (residential density, street connectivity, land use mix)	Individual: sex, age, education, employment status, occupation, living with children
Van Dyck, 2010 [43]	- Moderate-to-vigorous physical activity in minutes per day assessed with accelerometer (continuous) - Reported walking, recreation, and cycling for transport, and motorized transport in minutes per week (continuous)	Adults (20–65 years) N = 1,166	Belgium	Cross-sectional	Median annual household income	Walkability index (residential density, street connectivity, land use mix)	Individual: age, sex, education, working status, BMI
Wen, 2009 [47]	- Reported number of weekly workout/exercise (dichotomous): one to three times vs. four times or more - Reported regular exercise past year (dichotomous)	Adults (≥18 years) N = 3,530	USA	Cross-sectional	Index (households with an annual income >$50,000, families below the poverty line, residents ≥25 years with college education, female-headed households, households on public assistance, neighbourly trust, norms of reciprocity, violence)	Distance to subway and parks from the tract centroid; land use mix; number of art centres, cultural institutions, leisure venues, and entertainment facilities in a three-mile buffer from the tract centroid; number of restaurants and bars in a one mile-buffer; number of libraries, churches, and educational institutions in a two-mile buffer; number of health and human services in a three-mile buffer	- Individual: age, sex, ethnicity, marital status, education, household income - Contextual: pedestrian injuries per 100,000 persons, residential density
Inagami, 2009 [54]	BMI (continuous)	Adults (≥18 years) N = 2,156	USA	Cross-sectional	Index (residents below the poverty line, households headed by women, unemployed male residents, families on public assistance)	Number of fast food outlets and number of total food outlets divided by census tract roadway miles	Individual: age, sex, education, ethnicity, employment, marital status, annual household income, immigrant status, car ownership
Moore, 2013 [49]	BMI (continuous)	Adults (45–84 years) N = 1,503	USA	Cross-sectional	Index (Sixteen variables of education, occupation, income, and housing value)	Density of recreational facilities and healthy food environments in a one mile buffer around home address	Individual: age, sex, ethnicity, education, household income, perceived neighbourhood environment (aesthetic quality, walking environment, healthy food availability), perceived neighbourhood safety and social cohesion
Ross, 2007 [53]	BMI (continuous)	Adults (20–64 years) N = 32,964	Canada	Cross-sectional	Percentage of residents with low education, median household income	Dwelling density (dwellings per square kilometre)	- Individual: age, sex, income, education, marital status, smoking status, work-related physical activity, fruit/vegetable consumption, daily stress, immigrant status - Contextual: percentage of recent immigrants
Wang, 2007 [51]	BMI (continuous)	Adults (25–74 years) N = 7,595	USA	Cross-sectional	Index (median family income, median housing value, blue collar workers, unemployed residents, residents having less than high school education)	Total number of stores and fast food restaurants divided by neighbourhood size including a half mile buffer zone around the neighbourhood; proximity to food store or fast food restaurant from home address	Individual: age, sex, ethnicity, individual SEP index (household income and educational attainment), smoking status, physical activity, nutrition knowledge
Grafova, 2008 [50]	- Under-/normal weight vs. overweight/obesity (dichotomous) - Under-/normal weight vs. obesity (dichotomous)	Adults (≥55 years) N = 15,221	USA	Cross-sectional	- Index I (residents in poverty, residents ≥65 years in poverty, households on public assistance, unemployment rate of residents ≥16 years, housing units without a vehicle, black residents) - Index II (upper quartile value of owner-occupied housing units, families with annual income ≥$75,000, adults with a college degree)	Street connectivity; number of food stores, restaurants and housing units per square mile and by population density; PM10; summer ozone average	- Individual: age, sex, education, income, ethnicity, marital status, non-housing assets, region of birth, current region, self-rated health as a child, self-rated family SEP as a child, proxy response - Contextual: crime and segregation index, residential stability index, immigrant concentration
Wen, 2012 [52]	Under-/normal weight vs. obesity (dichotomous)	Adults (20–64 years) N = 9,739	USA	Cross-sectional	Index (households with annual income ≥$75,000, residents living in poverty, college-educated residents)	Street connectivity, distance to closest seven parks from tract centroid	- Individual: age, sex, education, ethnicity, poverty income ratio, smoking status, marital status, US-born - Contextual: Population density, ethnic heterogeneity, percentage of residents walking to work, age of neighbourhood buildings
	**Health outcomes and health related behaviours**						
Cummins, 2005 [65]	Self-rated health (dichotomous): very good or good health vs. bad or very bad health	Adults (≥16 years) N = 13,899	Britain and Scotland	Cross-sectional	Unemployment index based on claimant count	Food score (number of multiple owned food stores), bank and building society score (banks, building societies and automatic teller machines), health service score (pharmacies, opticians, general practitioners, dental practices), public recreation score (public swimming pools, libraries, and attendance at leisure centres), physical environment score (missed waste collections, public sector housing vacancy rate, vacant and derelict land)	- Individual: age, sex, social class, economic activity - Contextual: transport wealth score (high value cars), private transport score (number of cars per 1,000 population, number of private cars per 1,000 population, number of company cars per 1,000 population), political engagement score (voter turnout), left wing political climate score (political party in power), crime score (sexual/indecent crimes, violent offences, constables/special constables by police force area, spending on police services per capita)
Stafford, 2005 [69]	Self-rated health (dichotomous): very good or good health vs. bad or very bad health	Adults (≥16 years) N = 8,437	Britain and Scotland	Cross-sectional	Unemployment index based on claimant count	Food score (number of Tesco, Sainsbury and Safeway stores), bank and building society score (banks, building societies and automatic teller machines), health service score (pharmacies, opticians, general practitioners), public recreation score (public swimming pools, libraries, and attendance at leisure centres), physical environment score (public sector housing vacancy rate, vacant and derelict land)	- Individual: age, family type, sex, social class, economic activity - Contextual: transport wealth score (high value cars), private transport score (number of private cars per 1,000 population, number of company cars per 1,000 population), political engagement score (voter turnout), political climate score (left-wing political representation), crime score (reported sexual/indecent crimes, spending on police services per capita)
Freedman, 2011 [67]	Reported chronic diseases (dichotomous): heart problems, high blood pressure, stroke, diabetes, cancer, arthritis	Adults (≥55 years) N = 15,374	USA	Cross-sectional	-Index I (residents in poverty, residents ≥65 years in poverty, households receiving public assistance, unemployed residents ≥16 years, housing units without vehicle, black population) -Index II (upper quartile of the percentage of owner-occupied housing units, families with a total annual income of ≥$75,000, adults with a college degree)	Street connectivity, air pollution (PM10 and summertime ozone averages), density score (food stores, restaurants, housing units, and population density)	- Individual: age, sex, ethnicity, marital status, education, mean assets in $100,000, income, smoking, region of residence, region of birth, self-rated health and SEP during childhood, proxy response - Contextual: Immigrant concentration, crime and segregation, residential stability
Freedman, 2008 [68]	- Reported body limitations, such as climbing stairs, kneeling, crouching etc. (dichotomous) - Reported limitations of instrumental activities, such as shopping, cooking, etc. (dichotomous) - Reported limitations of daily living activities, such as bathing, dressing, eating etc. (dichotomous)	Adults (≥55 years) N = 15,480	USA	Cross-sectional	-Index I (residents in poverty, residents ≥65 years in poverty, households receiving public assistance, unemployed residents ≥16 years, housing units without a vehicle, black population) -Index II (upper quartile of the percentage of owner-occupied housing units, families with a total annual income of ≥$75,000, adults with a college degree)	Street connectivity, air pollution (PM10 and summertime ozone averages), density score (density of food stores, restaurants, housing units, and population density)	- Individual: age, sex, ethnicity, marital status, education, income, smoking, current region, region of birth, self-rated health and SEP during childhood, proxy response - Contextual: immigrant concentration, crime and segregation, residential stability
Matthews, 2010 [66]	Composite health score (continuous): presence of any of six physical health problems and self-rated health (higher values indicate better health)	Adults (≥18 years) N = 4,093	USA	Cross-sectional	- Index I (resident/room ratio, female-headed households, unemployment rate, poverty, people receiving public assistance) - Index II (residents with at least a bachelor´s degree, managerial or professional occupations)	Daily vehicle miles travelled, toxic release inventory sites, residual waste operations facilities, medical resources index (licensed and staffed beds, licensed medical doctors, hospitals, patients ≥65 years receiving flu vaccine)	- Individual: age, sex, ethnicity, stress level, marital status, employment status, retired, incapable of working, education, poverty status, religious service attendance, insurance and dental insurance, regular source of care, transportation difficulty in seeing a doctor, neighbourhood participation and trust - Contextual: residential stability, safety index (violent crimes, property crimes, missing persons)
Yang, 2010 [70]	Self-rated day-to-day stress on a scale from 1 to 10 (continuous): higher value indicate more stress	Adults (≥18 years) N = 4,095	USA	Cross-sectional	Index (female headed households, unemployment rate, poverty, residents receiving public assistance, median household income, residents with at least a bachelor´s degree)	Daily vehicle miles travelled based on length of road and average daily traffic estimate; toxic release inventory sites and residual waste operation sites	- Individual: age, sex, ethnicity, marital status, employment status, education, poverty, food insecurity, health score (calculated from reported physical health problems and self-rated health), religiosity, trust in neighbourhood people - Contextual: crime, residential stability
Dragano, 2009 [73]	Objective coronary artery calcification (dichotomous)	Adults (45–75 years) N = 4,301	Germany	Cross-sectional	Unemployment rate	Individual distance to major road from home address (>100 m and ≤100 m)	Individual: age, education
Dragano, 2009 [74]	Objective coronary artery calcification (dichotomous)	Adults (45–75 years) N = 4,301	Germany	Cross-sectional	Unemployment rate	Individual distance to major road from home address (0–50 m, 51–100 m, 101–200 m, ≥200 m)	Individual: age, education, economic activity, smoking, physical inactivity, overweight, hypertension, total cholesterol
Chuang, 2005 [71]	Reported number of smoked cigarettes on average per day (continuous)	Adults (25–74 years) N = 8,121	USA	Cross-sectional	Index (residents with less than high school education, blue collar workers, unemployed residents, median annual family income, median housing value)	Number of convenience stores per square mile, individual distance to nearest convenience store from home address, number of convenience stores in a one mile radius around home address	Individual: age, sex, ethnicity, individual SEP index (education, poverty status based on federal poverty threshold)
Pollack, 2005 [72]	Reported alcohol consumption (dichotomous): heavy alcohol consumption (>7 drinks per week for females; >14 drinks per week for males)	Adults (25–74 years) N = 8,197	USA	Cross-sectional	Townsend Material Deprivation Index (crowded occupied housing units, unemployed residents in the civilian labour force, tenant occupied housing units, occupied housing units without a vehicle available)	Number of alcohol outlets per square mile, distance to alcohol outlet from home address, number of alcohol outlets in a half mile radius around home address	Individual: age, sex, ethnicity, marital status, individual SEP index (income and education)
	**Perinatal and child health**						
Géneréux, 2008 [62]	- Preterm birth (dichotomous) - Low birth weight (dichotomous) - Small for gestational age (dichotomous)	Life births N = 99,819	Canada	Cross-sectional	Percentage of low-income families	Individual proximity to highway from home address (distance ≤200 m)	Individual: maternal age and education, infant´s sex, civil status, maternal country of birth, birth order, history of previous stillbirth, year of birth
Ponce, 2005 [63]	Preterm birth (dichotomous)	Life births N = 37,347	USA	Cross-sectional	Index (unemployed residents in the civilian labour force, households with public assistance income, families with income below the poverty line)	Distance-weighted traffic density based on individual distance to roadways from home address and annual average daily traffic counts	- Individual: maternal age, education, and ethnicity, payment for delivery, prenatal care, infant´s sex, parity, time since previous life birth, previous low birth weight or preterm infant, year of birth, live near highway, air pollutants - Contextual: season
Williams, 2007 [60]	Birth weight in grams (continuous)	Life births N = 13,559	USA	Cross-sectional	Percentage of residents below the poverty level	Average atmospheric concentration of sulphur dioxide, lead and fine particulates around infant´s home; number of hazardous waste sites in a 5 kilometre radius around infant´s home	Individual: maternal education and ethnicity, infant´s sex, previous infant delivery, previous infant >4,000 gram or <37 week, hypertension, oligohydramios, preeclampsia, previous non-live births, smoking, infants born from same pregnancy, other rare maternal risk factors
Zeka, 2008 [61]	- Birth weight in grams (continuous) - Small for gestational age (dichotomous) - Preterm birth (dichotomous)	Life births N = 425,751	USA	Cross-sectional	Median annual household income	Cumulative average daily traffic; individual distance to major highways from home address; percentage of open space designed for recreation, conversation, water supply, and forestry	Individual: age of mother, maternal education, ethnicity, prenatal visits, gestational age, smoking during pregnancy, previous infant greater than 4,000 gram, previous preterm birth, chronic or gestational conditions of mother, year of birth
Reading, 2008 [64]	- Reported number of child accidents by mother - Reported number of medical attended child injuries	Children (0–5 years) N = 41,409	Britain	Longi-tudinal	Percentage of unemployed residents, percentage of social classes 4 and 5	Road density of all roads, road density of major roads, percentage of detached and semi-detached housing, percentage of terraced housing, percentage of purpose built flats, percentage of converted flats	- Individual: child (age, sex, twin or triplet, ethnicity, physical activity, development, behavioural characteristics, motor functions, activity, risk avoidance, strength and difficulties, arguing with mother), mother (age, education, marital status, ethnicity, relationship status, partner moved out, lost partner, employment status, smoking status, alcohol and cannabis consume, depressive symptoms, significant life events, social support), partner (employment status, ethnicity, alcohol consumption), household (number and age of siblings, number of adults, lone parent, number of child caretakers, household income, financial difficulties, home owner, car), housing (rented, flat or room, garden, safety features), perceived neighbourhood environment (quality of neighbourhood, environmental problems, bus traffic, fear of crime, neighbourhood contacts), movement during data collection - Contextual: population aged 0–4 years, lone parents, movements, home owners, renters, overcrowded households, residents without a car, black population

Abbreviations:

SEP = Socioeconomic position; BMI = Body Mass Index; PM10 = quarterly measures of particulate matter at 10 μm or less
