# Supplementary material for: Interactive and Independent Associations between the Socioeconomic and Objective Built Environment on the Neighbourhood Level and Individual Health: A Systematic Review of Multilevel Studies
Source: PLoS One. 2015 Apr 7;10(4):e0123456. doi: 10.1371/journal.pone.0123456 (PMC4388459; doi:10.1371/journal.pone.0123456)
Supplement: S1 Text — (PDF) [file pone.0123456.s002.pdf]

PubMed (Advanced search) [1)]

(neighborhood [Title/Abstract] OR neighbourhood [Title/Abstract] OR area [Title/Abstract] OR place [Title/Abstract] OR residence [Title/Abstract] OR community [Title/Abstract] OR region [Title/Abstract]) AND (multilevel [Title/Abstract] OR multi-level [Title/Abstract] OR hierarch [Title/Abstract] OR "multilevel analysis" [MeSH Terms] OR "Small-Area Analysis" [MeSH Terms] OR "mixed effect*" [Title/Abstract] OR "random effect*" [Title/Abstract]) AND ("social environment*" [Title/Abstract] OR socioeconomic [Title/Abstract] OR socio-economic [Title/Abstract] OR sociodemographic [Title/Abstract] OR socio-demographic [Title/Abstract] OR "social environment" [MeSH Terms] OR "socioeconomic factors" [MeSH Terms]) AND ("physical environment*" [Title/Abstract] OR built [Title/Abstract] OR build* [Title/Abstract] OR "living environment*" [Title/Abstract] OR housing [Title/Abstract] OR pollution [Title/Abstract] OR burden* [Title/Abstract])*

PsycINFO (Expert search)

((neighborhood OR neighbourhood OR area OR place OR residence OR community OR region) AND (multilevel OR multi-level OR hierarch* OR "mixed effect*" OR "random effect*") AND ("social environment*" OR socioeconomic OR socio-economic OR sociodemographic OR socio-demographic) AND ("physical environment*" OR built OR build* OR "living environment*" OR housing OR pollution OR burden*)).ab,hw,id,ot,tc,ti,tm.

Web of Science (Advanced search)

TS=((neighborhood OR neighbourhood OR area OR place OR residence OR community OR region) AND (multilevel OR multi-level OR hierarch OR "mixed effect*" OR "random effect*") AND ("social environment*" OR socioeconomic OR socio-economic OR sociodemographic OR socio-demographic) AND ("physical environment*" OR built OR build* OR "living environment*" OR housing OR pollution OR burden*))*

1) Medical Subheadings were only available in PubMed and not in the two other databases
